# Effects of Heat-Killed *Levilactobacillus brevis* KB290 in Combination with β-Carotene on Influenza Virus Infection in Healthy Adults: A Randomized Controlled Trial

**DOI:** 10.3390/nu13093039

**Published:** 2021-08-30

**Authors:** Shohei Satomi, Naoko Waki, Chinatsu Arakawa, Kazuhiko Fujisawa, Shigenori Suzuki, Hiroyuki Suganuma

**Affiliations:** 1 Department of Nature & Wellness Research, Innovation Division, KAGOME Co., Ltd., 17 Nishitomiyama, Nasushiobara 329-2762, Japan; Naoko_Waki@kagome.co.jp (N.W.); Chinatsu_Arakawa@kagome.co.jp (C.A.); Shigenori_Suzuki@kagome.co.jp (S.S.); Hiroyuki_Suganuma@kagome.co.jp (H.S.); 2 Ageo Central Second Hospital, 421-1 Jitokata, Ageo, Saitama 362-0051, Japan; soumu@ach2.jp

**Keywords:** influenza virus, *Levilactobacillus brevis* KB290, β-carotene, combination effects, double-blind trial, healthy adults

## Abstract

Influenza, a seasonal acute respiratory disease caused primarily by the influenza virus A or B, manifests with severe symptoms leading to considerable morbidity and mortality and is a major concern worldwide. Therefore, effective preventive measures against it are required. The aim of this trial was to evaluate the preventive effects of heat-killed *Levilactobacillus brevis* KB290 (KB290) in combination with β-carotene (βC) on influenza virus infections in healthy Japanese subjects aged between 20 and 59 y throughout the winter season. We performed a randomized, double-blind, placebo-controlled, parallel-group trial from 16 December 2019 to 8 March 2020, comparing KB290 + βC beverage with placebo beverage. The primary endpoint was the incidence of influenza based on a doctor’s certificate. The incidence of influenza was not significantly different between the two groups. However, the subgroup analysis showed a significant difference between the two groups (influenza incidence: the KB290 + βC group 1.9%, and the placebo group 3.9%) in the subgroup of subjects aged ˂40 y, but not in the subgroup of subjects aged ≥40 y. The results of this trial suggest that the combination of KB290 and βC might be a possible candidate supplement for protection against the seasonal influenza virus infection in humans aged <40 y, although further clinical studies are needed to confirm the concrete preventive effect of this combination on influenza.

## 1. Introduction

Seasonal influenza is an acute respiratory disease that circulates periodically worldwide [1]. Influenza type A and B viruses (IAV and IBV, respectively) are a public health concern, causing clinical symptoms such as sudden onset fever, myalgia, headache, malaise, cough, sore throat, nasal congestion, and abdominal pain [2,3]. While vaccination against IAV and IBV is usually performed as a practical and prophylactic method [4], it may not be sufficient due to rapid viral mutagenesis [5]. Therefore, it is important to enhance host immune responses and resistance to influenza viruses in daily life.

Vitamins such as vitamin A (VA) or vitamin D have been studied for their effects on the immune system and immune function as well as their protective effects against influenza infection [6,7,8,9]. Lactic acid bacteria (LAB) have also been reported to exert immunomodulatory effects on the respiratory tract and protect the host against IAV infections [10,11]. However, the combined effects of these food components on influenza virus infections have rarely been investigated.

Our previous in vivo study demonstrated that the administration of heat-killed *Levilactobacillus brevis* KB290 (KB290) or retinoic acid, a metabolite of VA, suppressed weight loss and viral titer elevations in mice challenged with IAV [12]. In addition, the administration of KB290 in combination with retinoic acid significantly suppressed weight loss rather than KB290 or retinoic acid alone [12]. These findings suggest that this combination could be an effective prophylaxis for daily use.

However, excess intake of VA can lead to toxicity in humans [13], which hinders the conduction of human trials on VA consumption for influenza prevention. β-Carotene (βC), a pro-VA present in plant-derived foods such as carrots, is a safe candidate source of VA for human consumption. This is because VA production in humans from this component is regulated by an individual’s VA status [14]. No serious side effects of βC have been reported, except when a large amount of it was consumed as a supplement under high oxidative stress conditions like smoking [15]. Therefore, βC was selected in this trial since we considered that βC could contribute to maintaining adequate levels of VA/retinoic acid in human with safety.

We performed a randomized controlled trial to investigate whether a combination of KB290 and βC (KB290 + βC) could reduce the incidence of influenza and common colds as well as alleviate clinical symptoms in healthy Japanese adults. To our knowledge, this is the first study to demonstrate the combined effects of these food components on influenza infection in a large-scale human clinical trial.

## 2. Materials and Methods

### 2.1. Trial Design

We performed a randomized, double-blind, placebo-controlled, parallel-group trial from 16 December 2019 to 8 March 2020 in the Kanto (Tokyo, Kanagawa, Saitama, and Chiba), Hokkaido (Sapporo), and Kyushu (Fukuoka and Kumamoto) regions of Japan. This trial was approved by the Research Ethics Review Committee of Ageo Central Second Hospital and KAGOME Co., Ltd. (Approved number: IF1-1902 and 2019-R10, respectively) and registered with the University Hospital Medical Information Network Clinical Trial Registry (UMIN-CTR), with the ID number UMIN000038293. This study was conducted in accordance with the International Ethical Guidelines and the Helsinki Declaration. Written informed consent was obtained from all subjects prior to enrollment, and a background survey was conducted.

**Figure 1:**
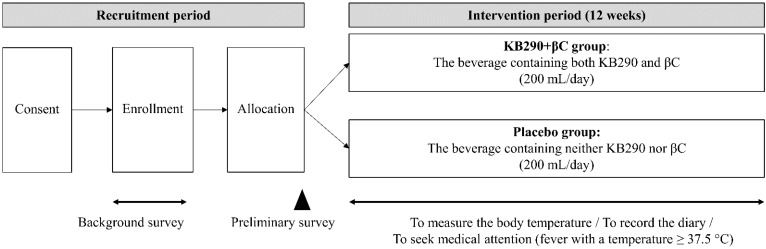
Trial design.

Figure 1 shows an outline of the trial design. After random allocation of the study’s subjects with permuted blocks, a preliminary survey was conducted on 15 December 2019. Then, each subject consumed one bottle (200 mL) of KB290 + βC beverage or a placebo beverage daily for 12 weeks (between 16 December 2019, and 8 March 2020). The allocation manager, who was not involved in this trial, created an allocation table and disclosed it until we completed data analysis in order to maintain blindness for the participants and researchers throughout the trial.

Intervention adherences in this trial were as follows: (1) The subjects were asked to maintain their lifestyle throughout the trial; (2) they recorded their doctor’s diagnosis (influenza or others) in the diary if they develop a fever (Temperature ≥ 37.5 °C) and had to seek medical attention; (3) they recorded diary every day; (4) they kept confidential about this trial; and (5) they were asked not to take pharmaceuticals, foods for specified health use, foods with functional claims, and other health foods. Other health foods in this trial meant the foods which are widely sold and used to improve and/or maintain health.

Their underarm body temperatures were measured at the same time point daily throughout the intervention period with a thermometer (OMRON HEALTHCARE Co., Ltd., product number: MC-141W-HP), but this time point was different from subject to subject. In addition to the body temperature, each subject recorded the degrees of all symptoms, including sore throat, cough, headache, and abdominal pain, in an electronic diary. For instance, if they developed a fever (Temperature ≥ 37.5 °C) and had to seek medical attention, they recorded their doctor’s diagnosis (influenza or others) in the diary.

### 2.2. Experimental Beverages

KB290 + βC and placebo beverages were manufactured by KAGOME Co., Ltd. One bottle of KB290 + βC drink had a volume of 200 mL and provided 67 kcal, 16.2 g carbohydrates, 0.7 g proteins, 0 g fat, at least 10^10^ cells of KB290, and 7.4–12.4 mg of βC. One bottle of placebo beverage also had a volume of 200 mL and provided 68 kcal, 16.2 g carbohydrates, 0.5 g proteins, and 0 g fat without KB290 or βC. The βC in the KB290 + βC beverages were derived from carrots and were not artificially added. The raw materials for the KB290 + βC drink were carrot juice, apple juice, KB290, and flavors. The raw materials for the placebo drink were β-carotene-reduced carrot juice, apple juice, and flavors. Both experimental beverages were in blue packages and undistinguishable.

### 2.3. Subjects

We recruited the subjects in this trial using the recruiting website (APO PLUS STATION Co., Ltd., Tokyo, Japan), and all subjects were Japanese residents. All subjects were healthy, aged between 20 and 59 y, and typically experienced the symptoms of colds at least once a year. The exclusion criteria were as follows: (1) those with a history of serious illness (liver/kidney/heart disease, organ damage, diabetes, food allergy disease, digestive disease, etc.); (2) those who regularly consumed lactic acid bacteria-fortified foods or vegetable juices (more than three times a week); (3) smokers; (4) those with a history of exposure to asbestos; (5) those with severe pollinosis; (6) pregnant and/or lactating women, including those who were planning a pregnancy during the study; (7) alcoholics; (8) those who routinely took pharmaceuticals, foods for specified health use, foods with functional claims, and other health foods; (9) those who were allergic to the experimental beverages; (10) those who could not take the experimental beverages as directed; (11) participants of other clinical studies; (12) those who suffered from influenza between 1 September 2019, and the date of obtaining written informed consent; (13) health workers.

Intake of βC at 20 mg/day for smokers [16] or at 30 mg/day for those with a history of asbestos exposure [17] has been reported to increase lung cancer morbidity. Therefore, in this study, both smokers and those with exposure to asbestos were excluded to eliminate the risk to increase lung cancer morbidity.

To determine our study sample size, we performed a simulation using information from previous influenza incidence rates and our previous clinical trial. Influenza morbidity is generally estimated to be 5–10% [18]. Here, we set the influenza incidence rate at the lower limit of 5% and calculated the sample size under the assumption that the KB290 + βC beverage would reduce influenza incidence by 50%. We calculated 986 as the number of subjects needed for each group. Considering potential dropouts, we set the initial numbers of each group to 1100.

### 2.4. Endpoints

The primary endpoints were: (1) influenza incidence; (2) fever (Temperature ≥ 37.5 °C) incidence; and (3) fever duration based on diary records and doctor’s certificates. The secondary endpoints were: (4) incidence and (5) degrees of each subjective symptom (sore throat, headache, cough, and abdominal pain). The degrees of each symptom upon the occurrence of influenza or fever were subjectively evaluated as normal, slight, moderate, or severe. Furthermore, (6) the period from fever onset to the return to normal temperature when influenza occurred, and (7) the maximum body temperatures during influenza or fever were also evaluated. 

The endpoints were calculated using the following formulas [19]:

Influenza incidence = total number of subjects who developed influenza/total number of subjects;(1)

Fever incidence = total number of subjects who developed a fever with a temperature of 37.5 °C or higher/total number of subjects;(2)

Fever duration = sum of the number of days the body temperatures were recorded as 37.5 °C or higher/total number of subjects who developed a fever with a temperature of 37.5 °C or higher;(3)

Incidence of subjective symptoms = total number of subjects who experienced symptoms (slight + moderate + severe)/total number of subjects(4)

Degree of subjective symptoms = (slight “1” × number of days slight symptoms were experienced + moderate “2” × number of days moderate symptoms were experienced + severe “3” × number of days severe symptoms were experienced)/(number of days slight symptoms were experienced + number of days moderate symptoms were experienced + number of days severe symptoms were experienced);(5)

Period before fever subsided (temperature below 37.5 °C) = sum of the number of days before return to normal temperature (below 37.5 °C) in the subjects who developed influenza/total number of subjects who developed influenza;(6)

Maximum body temperature = maximum body temperature of the subjects who developed influenza or a fever with a temperature of 37.5 °C or higher/number of subjects who developed influenza or a fever with a temperature of 37.5 °C or higher.(7)

### 2.5. Background Survey, Preliminary Survey, and Daily Questionnaire

Background surveys were performed on the same date as the signing of informed consent forms. These surveys asked for the following information: (1) gender; (2) date of birth; (3) age; (4) subjective symptoms experienced regularly; (5) medical history; (6) influenza vaccination history (including vaccination plans); (7) influenza infection from 1 September 2019 to the current date; 8) housemates under the age of 18; (9) occupation including their means of commute (ex. public transportation); and (10) inclusion and exclusion criteria.

Preliminary surveys were performed the day before the beginning of the trial (15 December 2019). This survey asked for the subject’s history of influenza vaccination and influenza incidence from the day informed consent was signed till the day before the intervention.

A questionnaire was administered once daily throughout the intervention period. This questionnaire asked for the following information: (1) intake status of the experimental beverages; (2) body temperature; (3) subjective four-level assessments (normal, slight, moderate, or severe) of each symptom (sore throat, headache, cough, and abdominal pain); (4) other subjective symptoms (optional); (5) hospitalization with specific details; (6) medications with their specific contents; and (7) influenza vaccination.

### 2.6. Safety Analysis

Safety analyses were performed on the subjects assigned to the experimental beverages at least once throughout the intervention period. The number of adverse events reported throughout the study period was determined. The principal doctor grasped the adverse events through the diary and/or direct offering from subjects. The principal doctor also evaluated for causal relationships of the adverse event with the experimental beverages.

### 2.7. Statistical Analyses

All statistical analyses were performed using SPSS version 25.0 software (IBM Japan, Ltd., Tokyo, Japan). The differences in influenza and fever incidence rates between the two groups were analyzed using the chi-square test. The differences in the other endpoints between the two groups were analyzed using the Wilcoxon rank-sum test. In all analyses, *p*-values ˂ 0.05 were considered statistically significant.

## 3. Results

### 3.1. Flowchart and Characteristics of the Trial Subjects

**Figure 2:**
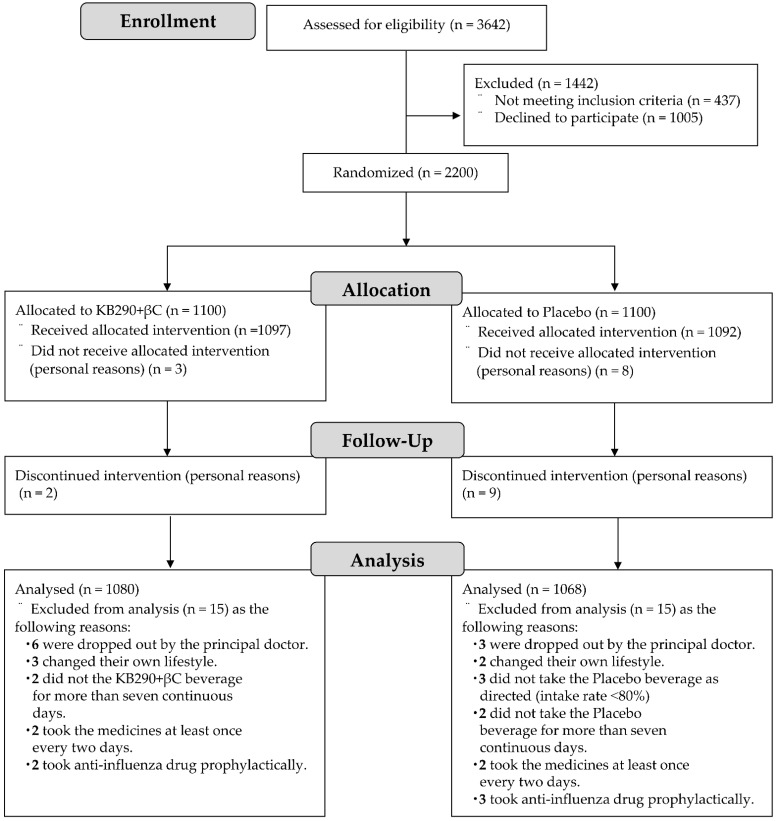
Flowchart of trial subjects. PP: per protocol analysis.

Figure 2 shows a flowchart of the subjects in this trial. The results of the preliminary survey showed that no subjects were infected with influenza prior to allocation. A total of 2200 subjects were enrolled in this trial and randomly allocated to the KB290 + βC group (1100 subjects) or the placebo group (1100 subjects). Of the 2200 subjects, 3 in the KB290 + βC group and 8 in the placebo group did not take the intervention for personal reasons, indicating that 1097 and 1092 in the KB290 + βC and placebo groups, respectively, took their respective experimental beverages at least once throughout the trial period. The KB290 + βC drink contained 6 × 10^10^ KB290 cells and 9.8 mg βC, as originally designed. Thus, safety analyses were performed on 2189 subjects.

Of these subjects, 2 in the KB290 + βC group and 9 in the placebo group discontinued intervention for their personal reasons. Six in the KB290 + βC group and 3 in the placebo group were dropped out by the principal doctor. Three in the KB290 + βC group and 2 in the placebo group changed their own lifestyle during this trial. Two in the KB290 + βC group and two in the placebo group did not take the experimental beverages for seven or more consecutive days; moreover, three in the placebo group did not take the placebo beverage as directed (adherence rate ˂ 80%). Two in the KB290 + βC group and two in the placebo group took the medicines at least once every 2 d. Furthermore, two in the KB290 + βC group and three in the placebo group received prophylactic anti-influenza drugs. Thus, efficacy analyses were performed on 2148 subjects (1080 in the KB290 + βC group and 1068 in the placebo group). The overall adherence rate to take the experimental beverages was 99.5%.

Table 1 shows the characteristics of the subjects studied for the efficacy analyses in this trial. No significant differences were identified between the two groups in any of the indices.

### 3.2. Primary and Secondary Endpoints

Influenza incidence, fever incidence, and fever duration were evaluated as the primary endpoints in this trial (Table 2). The rate of influenza incidence was 2.9% (31/1080) in the KB290 + βC group and 3.4% (36/1068) in the placebo group. There was no significant difference between the influenza incidences of the two groups (relative risk (RR), 0.852; 95% confidence interval (95% CI), 0.531–1.366; *p* = 0.51). Furthermore, the rate of fever incidence was 10.3% (111/1080) in the KB290 + βC group and 10.2% (109/1068) in the placebo group. There was no significant difference between the fever incidences of the two groups (RR, 1.007; 95% CI, 0.784–1.294; *p* = 0.96). Similarly, fever duration was not significantly different between the two groups (*p* = 0.33)—the average fever duration was 1.7 ± 1.2 d (mean ± SD) for the KB290 + βC group and 1.8 ± 1.0 d (mean ± SD) for the placebo group.

We evaluated the incidences and degrees of the subjective symptoms, durations until body temperatures returned to normal, and maximum body temperatures as the secondary endpoints. There were no significant differences between the two groups in any secondary endpoints (Tables S1–S4).

### 3.3. Subgroup Analysis

The study population was grouped according to sex, age, region, and influenza vaccination status. The influenza incidences in these subgroups were evaluated (Table 3).

In the subjects aged ˂40 y, the influenza incidence in the KB290 + βC group was significantly lower than that in the placebo group (1.9% (10/539) vs. 3.9% (21/538); RR: 0.465, 95% CI: 0.217–0.998, *p* = 0.044). However, in the subjects aged ≥40 y, the influenza incidence in the KB290 + βC group was not significantly different from that in the placebo group (3.9% (21/541) vs. 2.8% (15/530); RR: 1.387; 95% CI: 0.707–2.720; *p* = 0.34). Furthermore, in none of the other criteria grouped according to gender, area, or influenza vaccination, significant differences between the two groups were observed.

The fever incidence and fever duration were also investigated in the strata grouped according to age (Table 4).

In the subjects aged ˂40 years, the rate of fever incidence for the KB290 + βC group was significantly lower than that in the placebo group (9.5% (51/539) vs. 13.8% (74/538); RR: 0.655; 95% CI: 0.449–0.957; *p* = 0.028). In contrast, in the subjects aged ≥40 y, the fever incidence in the KB290 + βC group was significantly higher than that in the placebo group (11.1% (60/541) vs. 6.6% (35/530); RR: 1.764, 95% CI: 1.141–2.727, *p* = 0.010). 

In the subjects aged ˂40 y old, the average fever duration in the KB290 + βC group was significantly lower than that in the placebo group (1.5 ± 1.0 d vs. 1.7 ± 0.9 d (mean ± SD); *p* = 0.018), whereas in the subjects aged ≥40 y, the average fever duration in the KB290 + βC group was not significantly different from that in the placebo group (2.0 ± 1.2 d vs. 1.8 ± 1.2 d (mean ± SD); *p* = 0.66).

### 3.4. Safety Analysis

Adverse events, including subjective symptoms (sore throat, headache, cough, and abdominal pain) and other symptoms based on the diary records, were reported at least once throughout the intervention period in 859/1097 subjects in the KB290 + βC group (78.3%) and 829/1092 subjects in the placebo group (75.9%). Six serious adverse events (gallstones, ovarian cysts, fracture, colorectal polyps, urethra stones, and hospitalization for cervical hardening) were reported, but the principal doctor concluded that there were no causal relationships between these serious adverse events and the experimental beverages.

## 4. Discussion

We showed that the consumption of KB290 + βC did not significantly reduce influenza incidence, fever incidence, or incidence/degree of clinical symptoms in the subjects in this trial. However, we provided evidence that the experimental beverage significantly reduced influenza incidence in the subjects aged ˂40 y, without any associated serious adverse events.

We estimated the influenza incidence in the placebo group in this trial to be 5% since this was the assumed lowest influenza incidence during the sample size calculation. However, the incidence of influenza throughout the winter season (2019–20) in Japan was lower than in any of the last five winter seasons (from 2014–15 to 2018–19) [20], and the actual rate in the placebo group was 3.4%. The lack of significant differences in the analysis of all subjects in this trial could be attributed to this. 

Furthermore, the lower influenza incidence in 2019–20 could also be due to the outbreak of the new coronavirus disease 2019 (COVID-19) [21]. The first case of COVID-19 infection in Japan was reported on January 15, 2020, during the intervention period of this trial [22,23]. Due to this, the Ministry of Health, Labour and Welfare of Japan (MHLW) asked the public to adhere to cough etiquette and handwashing [24,25,26]. Measures taken against COVID-19 may also have been effective in preventing influenza infection [1]. In addition, the warmer temperature during this season could also have contributed to the lower incidence of influenza, as indicated by a previous study reporting that influenza activity could be affected by temperature [27]. In fact, the average temperature in the winter season (from January to March in 2020) in Hokkaido, Kanto, and Kyushu was >1 °C higher than usual (data not shown). Taken together, this winter season might not have been suitable for the evaluation of the preventive effects of KB290 + βC on influenza due to its low prevalence.

Despite these conditions, the subgroup analysis on the subjects aged ˂40 y showed that KB290 + βC significantly reduced influenza incidence. This could be due to low awareness of prevention against infection and low βC intake from the daily diets of subjects aged ˂40 y. An internet survey of Japanese citizens’ behavioral changes against COVID-19 showed that people aged 40–59 practiced social distancing to prevent infectious diseases more than people aged 20–29 [28]. Practicing social distancing was also effective in controlling influenza outbreaks [29]. This means that the subjects aged ˂40 y were more likely to be exposed to the influenza virus than those aged ≥40 y. In fact, in the placebo group, the influenza incidence in those aged ˂40 y was higher than that in those aged ≥40 y (3.9% vs. 2.8%). However, the subjects in this trial did not record how often they practiced social distancing in their diary. Therefore, it was unclear whether the subjects ˂40 y old were more exposed to infection than the ≥40 y old subjects.

Furthermore, a previous cross-sectional study in Japan reported lower vegetable consumption and blood βC levels in those aged ˂40 y than in those aged ≥40 y [30]. In addition, the National Health and Nutrition Survey in 2019 conducted by MHLW shows that the average intake of VA in those aged 20–29 years, 30–39 years, 40–49 years, or 50–59 years was 449, 438, 504, or 536 μgRE (retinol equivalent)/day/person, respectively. This survey indicated those aged <40 y took less VA than those aged ≥40 [31]. Similarly, *Dietary Reference Intakes for Japanese* (2020) proposed by MHLW shows that the estimated average requirement in VA is 600–650 μgRE/day/person for males and 450–500 μgRE/day/person for females [32]. This means that the VA intake of many young Japanese does not reach the estimated average requirement. Though neither vegetable intake nor blood βC levels in the subjects were measured in this trial, these may be lower in the subjects aged ˂40 y than those in the subjects aged ≥40 y. It can be inferred that the effect of βC on influenza infection may be more pronounced in subjects with lower vegetable intake and blood βC levels than in those with higher levels of these. Therefore, the effectiveness of βC on the subjects aged ˂40 y against influenza, even in a low prevalence setting, could be attributed to this discrepancy in the vegetable intake and blood βC levels. 

Collectively, KB290 + βC may have a protective effect against influenza infection in those at high risk of infection and more likely to benefit from βC supplementation. However, the subgroup analysis on the subjects aged ≥40 y demonstrated that the fever incidence in the KB290 + βC group was significantly higher than that in the placebo group. A fever with a temperature of 37.5 °C or higher was set as the index to evaluate the effects of KB290 + βC on common colds, including influenza infection. However, some subjects who developed common colds as diagnosed by their doctors did not develop fevers. In addition, it was confirmed that fever not only develops in colds and influenza infections but also in unknown causes, in which no description of these subjective symptoms was recorded in the diary. In the subgroup aged ≥40 y, we focused on the subjects who developed a fever. Twenty out of 60 subjects in the KB290 + βC group and 8 out of 35 subjects in the placebo group developed fevers derived from causes other than common colds or influenza, such as gastroenteritis, joint pains, and mumps, including unexplained fever. Collectively, these findings suggest that fever incidence was not an appropriate index for evaluating the effects of KB290 + βC on the common cold in this trial.

The strength of the present trial lies in using a large-scale randomized controlled design in healthy adults. Previous studies focusing on the preventive effects of food components on influenza have methodological limitations, including inadequate study design [33], limited diversity of the subjects [34,35], and small-scale size [19]. Despite subdivisions of the study population into two groups according to age, each group still had more than 500 subjects, eliminating the risk of random effects unrelated to the intervention. 

Nevertheless, the present trial has several limitations. First, this trial has a lack of information on the mechanism of KB290 + βC against influenza, as we did not measure immunological parameters such as innate immune responses or antibodies in this trial. Our previous studies have shown that live KB290 induced the production of interferon-α and immunoglobulin A in mice challenged with IAV [36] and enhanced cell-mediated cytotoxic activity in mice [37]. In addition to the immunoregulatory actions of βC [38], retinoic acid has also been reported to be associated with retinoic acid-inducible gene-I-mediated production of interferon-α and immunoglobulin A [39]. Future studies should investigate the mechanisms of KB290 + βC in the suppression of the influenza virus by determining its effects on immunological parameters in healthy adults and also investigate why and how KB290 and/or βC work(s) against influenza infection employing influenza-challenged model mice. Second, this trial also has a lack of information on nutritional status and dietary intakes. It was unclear whether blood βC levels in the subjects aged ˂40 y were actually low before the intervention in this study and whether our intervention increased blood βC levels in them. In the future, we should also investigate the relationship between blood βC levels and influenza incidence. Third, the analysis focusing on the age (over and under 40 y) in this trial was post-planned after the intervention finished, and we did not consider the multiple comparisons. Therefore, we should further investigate the effects of the KB290 + βC on the subjects aged ˂ 40 y in order to conclude the preventive effects of these combinations against influenza.

## 5. Conclusions

In conclusion, the results of this trial suggest that KB290 in combination with βC might be a possible candidate for protection against seasonal influenza virus infections in humans aged <40 y, although further clinical studies are needed to confirm the concrete preventive effect of this combination on influenza.

## Figures and Tables

**Table 1:** Characteristics of the subjects evaluated with efficacy analyses in this trial ^1^.

Variable		Total (*n* = 2148)	KB290 + βC (*n* = 1080)	Placebo (*n* = 1068)	*p*-Value
Age	20–29	572	283	289	0.44
	30–39	505	256	249	
	40–49	642	337	305	
	50–59	429	204	225	
	Average age	38.7 ± 11.1	38.7 ± 11.0	38.7 ± 11.2	
Sex	Female/male	1141/1007 (53.1%)	570/510 (52.8%)	571/497 (53.5%)	0.75
Region	Hokkaido	720	360	360	1.0
	Kanto	719	360	359	
	Kyushu	709	360	349	
Influenza vaccine (including plans)	Yes/No	1011/1137 (47.1%)	506/574 (46.9%)	505/563 (47.3%)	0.84
Residing with children aged <18 years	Yes/No	786/1362 (36.6%)	395/685 (36.6%)	391/677 (36.6%)	0.99
Daily use of public transportation	Yes/No	852/1296 (39.7%)	427/653 (39.5%)	425/643 (39.8%)	0.90

^1^ Values are expressed as the number of subjects except for the average ages (mean ± SD; standard deviations), which are expressed as age. The percent sign (%) indicates the rate of “female” or “Yes.” All *p*-values were computed using the chi-square test between the two groups.

**Table 2:** Influenza incidence, fever incidence, and fever duration ^1^.

	KB290 + βC	Placebo	RR	95% CI	*p*-Value
Influenza incidence	31/1080 (2.9%)	36/1068 (3.4%)	0.852	0.531; 1.366	0.51
Fever incidence	111/1080 (10.3%)	109/1068 (10.2%)	1.007	0.784; 1.294	0.96
Fever duration	1.7 ± 1.2	1.8 ± 1.0	-	-	0.33

^1^ Values in “influenza incidence” and “fever incidence” are expressed as incidence/total number of subjects. The percent sign (%) indicates the rate of influenza or fever incidence. Values in “fever duration” are expressed as average days (mean ± SD).

**Table 3:** Influenza incidence as the primary endpoint, sub-grouped according to factors ^1^.

	KB290 + βC	Placebo	RR	95% CI	*p*-Value
Sex					
Female	16/570 (2.8%)	20/571 (3.5%)	0.796	0.408; 1.552	0.50
Male	15/510 (2.9%)	16/497 (3.2%)	0.911	0.445; 1.863	0.80
Age					
˂40 years	10/539 (1.9%)	21/538 (3.9%)	0.465	0.217; 0.998	0.044
≥40 years	21/541 (3.9%)	15/530 (2.8%)	1.387	0.707; 2.720	0.34
Region					
Hokkaido	9/360 (2.5%)	16/360 (4.4%)	0.551	0.240; 1.264	0.15
Kanto	13/360 (3.6%)	12/359 (3.3%)	1.083	0.487; 2.408	0.84
Kyushu	9/360 (2.5%)	8/349 (2.3%)	1.093	0.417; 2.866	0.86
Influenza vaccination status					
Vaccinated	15/434 (3.5%)	12/459 (2.6%)	1.334	0.617; 2.882	0.46
Unvaccinated	16/646 (2.5%)	24/609 (3.9%)	0.619	0.326; 1.177	0.15

^1^ Values in “influenza incidence” are expressed as incidence/total number of subjects. The percent sign (%) indicates the rate of influenza incidence.

**Table 4:** Fever incidence and fever duration as the primary endpoints, sub-grouped according to age ^1^.

	KB290 + βC	Placebo	RR	95% CI	*p*-Value
Fever incidence					
Aged ˂ 40 years	51/539 (9.5%)	74/538 (13.8%)	0.655	0.449; 0.957	0.028
Aged ≥ 40 years	60/541 (11.1%)	35/530 (6.6%)	1.764	1.141; 2.727	0.010
Fever duration					
Aged ˂ 40 years	1.5 ± 1.0	1.7 ± 0.9	-	-	0.018
Aged ≥ 40 years	2.0 ± 1.2	1.8 ± 1.2	-	-	0.66

^1^ Values in “fever incidence” are expressed as incidence/total number of subjects. The percent sign (%) indicates the rate of fever incidence. Values in “fever duration” are expressed as average days (mean ± SD).

## Data Availability

De-identified data described in the article, code book, and analytic codes will be made available upon request pending approval of an application for data use, and execution of a data use agreement and/or material transfer agreement with KAGOME Co., Ltd.

## Supplementary Materials

The following are available online at https://www.mdpi.com/article/10.3390/nu13093039/s1, Table S1: Incidence and degree of typical subjective symptoms during influenza infection, Table S2: Incidence and degree of typical subjective symptoms during fever, Table S3: Duration until body temperature returned to normal and maximum body temperature during influenza infection, and Table S4: Maximum body temperature during fever.
