# Supplementary material for: Effects of Heat-Killed Levilactobacillus brevis KB290 in Combination with β-Carotene on Influenza Virus Infection in Healthy Adults: A Randomized Controlled Trial
Source: Nutrients. 2021 Aug 30;13(9):3039. doi: 10.3390/nu13093039 (PMC8467669; doi:10.3390/nu13093039)
Supplement: Supplementary file 1 [file nutrients-13-03039-s001.zip › nutrients-1303183-supplementary.pdf]

Satomi S. *et al.*, Effects of heat-killed *Levilactobacillus brevis* KB290 in combination with β-carotene on influenza virus infection in healthy adults: a randomized controlled trial: Supplementary Files.

Table S1–S4.

Table S1. Incidence and degree of typical subjective symptoms during influenza infection [1].

Typical symptoms		KB290+βC (n = 31)	Placebo (n = 36)	*p*-value
Incidence	Sore throat	26/5	32/4	0.72
	Headache	29/2	33/3	1.0
	Cough	31/0	34/2	0.50
	Abdominal pain	11/20	8/28	0.23
Degree	Sore throat	1.38 ± 0.41	1.28 ± 0.33	0.43
	Headache	1.57 ± 0.49	1.47 ± 0.40	0.47
	Cough	1.42 ± 0.45	1.30 ± 0.30	0.33
	Abdominal pain	1.11 ± 0.19	1.29 ± 0.70	1.0

[1] Values in "Incidence" are expressed as yes/no numbers of subjects. Values in "Degree" are expressed as average scores (mean ± SD).

Table S2. Incidence and degree of typical subjective symptoms during fever [1].

Typical symptoms		KB290+βC (n = 111)	Placebo (n = 109)	*p*-value
Incidence	Sore throat	69/42	74/35	0.37
	Headache	81/30	83/26	0.59
	Cough	75/36	67/42	0.34
	Abdominal pain	34/77	31/78	0.72
Degree	Sore throat	1.70 ± 0.73	1.61 ± 0.68	0.53
	Headache	1.55 ± 0.67	1.63 ± 0.63	0.29
	Cough	1.51 ± 0.57	1.45 ± 0.54	0.49
	Abdominal pain	1.40 ± 0.65	1.40 ± 0.66	0.87

[1] Values in "Incidence" are expressed as yes/no numbers of subjects. Values in "Degree" are expressed as average scores (mean ± SD).

Table S3. Duration until body temperature returned to normal and maximum body temperature during influenza infection [1].

	KB290+βC (n = 31)	Placebo (n = 36)	*p*-value
Duration until body temperature returned to normal	2.0 ± 0.9	2.0 ± 1.1	0.94
Maximum body temperature	38.72 ± 0.64	38.61 ± 0.76	0.84

[1] Values in "Duration until body temperature returned to normal" are expressed as average days (mean ± SD). Values in "Maximum body temperature" are expressed as average maximum body temperatures (mean ± SD).

Supplementary Table S4. Maximum body temperature during fever [1].

	KB290+βC (n = 111)	Placebo (n = 109)	*p*-value
Maximum body temperature	38.22 ± 0.67	38.29 ± 0.66	0.26

[1] Values in "Maximum body temperature" are expressed as average maximum body temperatures (mean ± SD).
